# The effects of small-scale, homelike facilities for older people with dementia on residents, family caregivers and staff: design of a longitudinal, quasi-experimental study

**DOI:** 10.1186/1471-2318-9-3

**Published:** 2009-01-20

**Authors:** Hilde Verbeek, Erik van Rossum, Sandra MG Zwakhalen, Ton Ambergen, Gertrudis IJM Kempen, Jan PH Hamers

**Affiliations:** 1 School for Public Health and Primary Care, Caphri, Faculty of Health, Medicine and Life Sciences, Department of Health Care and Nursing Science, Maastricht University, Maastricht, the Netherlands; 2 School for Public Health and Primary Care, Caphri, Faculty of Health, Medicine and Life Sciences, Department of Methodology and Statistics, Maastricht University, Maastricht, the Netherlands

## Abstract

**Background:**

Small-scale and homelike facilities for older people with dementia are rising in current dementia care. In these facilities, a small number of residents live together and form a household with staff. Normal, daily life and social participation are emphasized. It is expected that these facilities improve residents' quality of life. Moreover, it may have a positive influence on staff's job satisfaction and families involvement and satisfaction with care. However, effects of these small-scale and homelike facilities have hardly been investigated. Since the number of people with dementia increases, and institutional long-term care is more and more organized in small-scale and homelike facilities, more research into effects is necessary. This paper presents the design of a study investigating effects of small-scale living facilities in the Netherlands on residents, family caregivers and nursing staff.

**Methods and design:**

A longitudinal, quasi-experimental study is carried out, in which 2 dementia care settings are compared: small-scale living facilities and regular psychogeriatric wards in traditional nursing homes. Data is collected from residents, their family caregivers and nursing staff at baseline and after 6 and 12 months of follow-up. Approximately 2 weeks prior to baseline measurement, residents are screened on cognition and activities of daily living (ADL). Based on this screening profile, residents in psychogeriatric wards are matched to residents living in small-scale living facilities. The primary outcome measure for residents is quality of life. In addition, neuropsychiatric symptoms, depressive symptoms and social engagement are assessed. Involvement with care, perceived burden and satisfaction with care provision are primary outcome variables for family caregivers. The primary outcomes for nursing staff are job satisfaction and motivation. Furthermore, job characteristics social support, autonomy and workload are measured. A process evaluation is performed to investigate to what extent small-scale living facilities and psychogeriatric wards are designed as they were intended. In addition, participants' satisfaction and experiences with small-scale living facilities are investigated.

**Discussion:**

A longitudinal, quasi-experimental study is presented to investigate effects of small-scale living facilities. Although some challenges concerning this design exist, it is currently the most feasible method to assess effects of this relatively new dementia care setting.

## Background

It is estimated that around 24 million people suffered from dementia worldwide in 2001 and this number will double every 20 years [1]. Most people suffering from dementia are cared for at home, but institutional care is often inevitable as the disease progresses. Institutional dementia care is increasingly organized in small-scale and homelike facilities. These are facilities in which a small number of residents live together in a homelike environment. Normalization of daily life with person-centered care is a central theme in these facilities [2]. In the literature, this care concept is also referred to as a 'home' model [3] or 'housing' model [4] as opposed to the medical model of care. Traditionally, institutional care for people with dementia has been organized to this medical model [5,6] and this has resulted in large-scale institutional nursing homes. Nowadays, policy principles emphasize that institutional care should be as homelike as possible [7]. Small-scale and homelike facilities are the result of this shift in dementia care concept. Differences with traditional nursing homes exist at a physical, social and organizational level. Table 1 presents a summary of main differences [3,8-10].

**Table 1:** Physical, social and organizational characteristics: traditional nursing homes vs. small-scale living facilities.

	Traditional nursing home	Small-scale living facility
Physical	- Large-scale wards (>20 residents) - Long corridors - Institutional character	- Small units (6 – 8 residents) - Homelike character, based on a archetype house

Social	- Many fellow residents and nursing staff working at one ward	- Emphasis on family situation - Residents form a group - Nursing staff is part of the household

Organizational	- more 'Top-down': organization/nursing home decides daily routine - Task-differentiation: many different functions and staff	- more 'bottom-up': residents and family caregivers have a large influence on daily routine - Nursing staff have integrated tasks: i.e. medical, personal care, activities and household

In many countries small-scale and homelike facilities have been established, such as group living in Sweden [11], Green Houses in the United States [12] and residential groups in Germany [13]. In the Netherlands, there is nowadays a large increase of small-scale living facilities, also referred to as group living [10]. It is expected that in 2010, approximately 25% of Dutch nursing home care for older people with dementia is organized in small-scale living facilities. In Sweden, almost 20% (14,000) of people with dementia residing in institutional care lived in group living facilities in 2000 [14].

Despite these developments, little is known yet about effects of a small-scale and homelike environment on residents, family and professional caregivers [2]. Some studies [15-18] report positive findings for residents. It is suggested that residents in small and homelike facilities have a better mobility [15], more social capacities [16] and a higher quality of life [17,18] than residents living in traditional nursing homes. However, more behavioral problems have also been reported for residents in small, homelike facilities [19]. Family members in small-scale living facilities appear to be more satisfied with care [20] and seem to experience less burden than family in traditional nursing homes [21]. Findings from staff members indicate that they may have a higher job satisfaction and motivation than in traditional nursing home care [22,23], although negative results such as a higher workload have also been reported [22].

Most studies regarding the effects of small-scale living facilities for older people with dementia suffer from several methodological limitations, such as inclusion of a small number of residents [15], no follow-up measurements [19], differences at baseline between residents in small-scale living and traditional nursing home care [17] or no control group at all. These drawbacks limit the interpretation of results. Since the number of people with dementia will increase worldwide [1,24] and dementia care will be more and more organized in small-scale and homelike facilities, more research and knowledge regarding effects of this environment is necessary.

### Aim and research questions

The current paper presents the design of a Dutch longitudinal, quasi-experimental study, investigating the effects of small-scale living facilities for older people with dementia. Residents, their family and nursing staff of small-scale living facilities are compared with those living in regular psychogeriatric wards of traditional nursing homes on several outcome measures. The three research questions are:

1. What are the effects of small-scale living facilities on residents' quality of life, behavioral problems and social engagement?

2. What are the effects of small-scale living facilities on family caregivers' involvement, satisfaction with care delivery and perceived burden from informal care?

3. What are the effects of small-scale living facilities on staff's job satisfaction, motivation and work perception, such as perceived social support, autonomy and burden?

In addition, a process evaluation is performed with 2 main goals: 1) to investigate to what extent both types of dementia care settings are designed as they were intended and 2) to investigate participants' satisfaction and experience with small-scale living facilities.

## Methods and design

**Figure 1:**
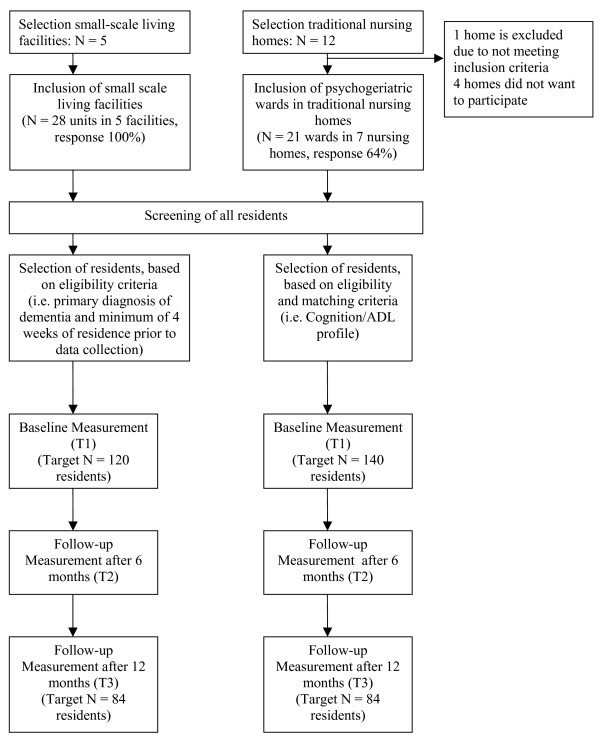
**Flow chart design and measurements**.

A longitudinal, quasi-experimental study is carried out (April 2008 – January 2010). Two types of dementia care settings are compared: small-scale living facilities (experimental group) and psychogeriatric wards in traditional nursing homes (control group). Outcome measures regarding residents, family care givers and nursing staff are measured at three moments in time: a baseline measurement (T1) and after 6 (T2) and 12 months (T3) after baseline. To enhance comparability of groups at baseline, residents are matched, using a screening procedure approximately 2 weeks prior to T1. Figure 1 presents a flow chart of the design and data collection. In addition to the effect study, a process evaluation is performed.

The study design and protocols are approved by the Medical Ethics Committee of the University Hospital Maastricht and Maastricht University. In addition, local Ethical Committees of participating institutions have given their consent to the study protocols and procedures.

### Target population

The target populations of this study are older people with dementia, who receive institutional nursing home care, their family caregivers and nursing staff working at their unit. They are recruited in two types of dementia care settings: small-scale living facilities and psychogeriatric wards in traditional nursing homes, all in the southern part of the Netherlands.

#### Residents

All residents in small-scale living facilities are eligible for participation in this study, if they 1) have a primary diagnosis of dementia, based on criteria established by the *Diagnostic and Statistical Manual of Mental Disorders*, fourth edition [25] and 2) have been living in the care setting for at least 4 weeks prior to data collection. The type and severity of the dementia syndrome may vary. Residents with a primary psychiatric disease or those with Korsakoff's syndrome are excluded, because they usually differ from other residents with dementia (e.g. have a better mobility and are younger) and live often in special wards. Residents living in psychogeriatric wards in traditional nursing homes are eligible if they meet the above mentioned criteria and in addition match the cognition and ADL-profile of residents in small-scale living facilities. This is assessed in a screening prior to the baseline measurements.

#### Family caregivers

A family caregiver is in this study defined as someone who has or takes the responsibility for a resident with dementia at a voluntary basis. All main family caregivers providing informal care for participating residents in this study are eligible. The number is limited to one main family caregiver per resident.

#### Staff

All nursing staff (i.e. nursing assistants, certified nursing assistants and registered nurses) working on a permanent basis in either the selected small-scale living facilities or regular psychogeriatric wards in which the residents live are eligible to participate in the study. Temporary staff, such as trainees, are excluded from the study.

### Small-scale living facilities: experimental group

Small-scale living facilities had to fulfill the following criteria to be eligible for this study:

1. A maximum of 8 residents per house or unit. This number is considered in the Netherlands as a maximum number for small-scale living [10].

2. Staff, residents and their family form a household together: activities are centered around the daily life and household. An important requirement is that staff prepare all meals together with residents and/or their family caregivers.

3. Staff perform integrated tasks: this means that one person may fulfill multiple tasks such as medical and personal care, domestics chores and activities.

4. Residents are cared for by a small, fixed team of professional caregivers, which are part of the household.

5. Daily life is organized completely or in a large amount by residents, their family caregivers and nursing staff.

6. Archetype home: a physical setting that resembles a homelike environment.

These criteria are based on a concept map, designed by te Boekhorst et al. (2007) [10] and on characteristics as presented in Table 1.

### Psychogeriatric wards in traditional nursing homes: control group

In the Netherlands, usual care for older people with dementia consists of care in psychogeriatric wards in traditional nursing homes. Inclusion criteria for these wards are:

1. A minimum of 20 residents per ward.

2. Staff have differentiated tasks: their main tasks entail medical and personal care for residents. Other tasks, such as domestic chores and (social) activities are provided by other specialized disciplines.

3. Residents and their family members have little control over the organization of daily life within the ward. Daily life is mainly organized around the routines of the nursing home.

### Measures

Table 2 presents all outcome and additional measures, their operationalization and timing of measurements.

**Table 2:** Data collection: outcome, operationalization and time of measurement.

Outcome measure	Operationalization	Time of measurement			
**Residents**		**S**	**T1**	**T2**	**T3**

*Primary outcome*					

Quality of Life	QUALIDEM [26-28]		Q	Q	Q

*Secondary outcome*					

Neuropsychiatric symptoms	NPI-NH [30-33]		Q	Q	Q

	CMAI [34,35]		Q	Q	Q

Depression symptoms	CSDD [36,37]		Q	Q	Q

Social Engagement	Subscale ISE and RISE form RAI MDS [38,39]		Q	Q	Q

*Additional variables*					

ADL-capacity	Subscale ADL-H from RAI MDS [40,41]	SQ		Q	Q

Cognition	MMSE [42]		Q		

	Subscale CPS from RAI MDS [40,43]	SQ		Q	Q

Use of physical restraint	Number of times physical restraints are used		Q	Q	Q

Psychotropic medication	ATC classification system [44]		Q	Q	Q

Use of health care services	Visits to e.g. Nursing home physician, psychologist etc.		Q	Q	Q

Dementia type	Alzheimer's dementia Vascular dementia, Other (e.g. Parkinson's disease)		MR		

Stage of dementia	GDS [29]		Q	Q	Q

Comorbidity	International classification of diseases, version 10 [46]		MR	MR	MR

*Socio-demographic variables*					

Age	Years	SQ			

Gender	Male or Female	SQ			

					

Length of Stay	Number of months	SQ			

Living prior to admission	At home, Residential care, Regular Nursing home care, Other	SQ			

**Family caregivers**					

*Primary outcome*					

Involvement with care	Frequency, length, activities and motivation for visits		Q	Q	Q

Perceived burden	SPPIC [47]		Q	Q	Q

Satisfaction with care	27 items		Q	Q	Q

*Additional variables*			Q	Q	Q

Age	Years		Q	Q	Q

Gender	Male or Female		Q	Q	Q

Sense of competence	SSCQ [49]		Q	Q	Q

Relationship with resident	E.g. Spouse, Child, Sibling or Other		Q	Q	Q

**Nursing staff**			Q	Q	Q

*Primary outcome*			Q	Q	Q

Job satisfaction & motivation	[45]		Q	Q	Q

*Secondary outcome*			Q	Q	Q

Social support	Subscale from JCQ [51,52]		Q	Q	Q

Autonomy	MAQ [50]		Q	Q	Q

Workload	[45]		Q	Q	Q

*Additional variable*					

Age	Years		Q	Q	Q

Gender	Male or Female		Q	Q	Q

Education level	Type of education and level (e.g. level 1 – 5)		Q	Q	Q

Contract working hours	Hours		Q	Q	Q

Years of employment	Years		Q	Q	Q

S = Screening, approximately one week prior to baseline measurement

T1 = Baseline measurement

T2 = Follow-up after 6 months

T3 = Follow-up after 12 months

Q = Questionnaire, SQ = Screening Questionnaire, MR = Medical Record

#### Residents

The primary outcome measure for residents is quality of life (QoL), as assessed by the QUALIDEM [26-28]. The QUALIDEM is a dementia-specific QoL instrument, developed for use in residential care and is rated by professional caregivers or proxies. It is a multi-dimensional scale and consists of 37 items, divided in 9 homogeneous subscales: Care relationship (7 items), Positive affect (6 items), Negative affect (3 items), Restless tense behavior (3 items), Positive self image (3 items), Social relations (6 items), Social isolation (3 items), Feeling at home (4 items) and Having something to do (2 items). Of these subscales, 6 can be used in very severe dementia (Global Deterioration Scale stage 7 [29]) using approximately half of the items [26]. Items describe observable behaviors present last week and comprises 4 response options each: never, seldom, sometimes and often. The reliability (coefficient Rho .60 – .90) and validity are found to be appropriate for evaluation of interventions [26,27].

Secondary outcome measures are: neuropsychiatric symptoms (Neuropsychiatric Inventory, Nursing Home version (NPI-NH) [30-33] and Cohen-Mansfield Agitation Inventory (CMAI) [34,35]), depressive symptoms (Cornell Scale for Depression in Dementia (CSDD) [36,37]) and social engagement (Index for Social Engagement (ISE), a subscale form the Resident Assessment Instrument (RAI) Minimum Data Set (MDS, version 2.1) [38] and Revised Index for Social Engagement (RISE) [39]).

Furthermore, several health-related variables are measured: ADL-capacity (ADL-Hierarchy (ADL-H), a subscale from the RAI-MDS (versions 2.1) [40,41]), cognition (standardized Mini-Mental State Examination (MMSE) [42] and Cognitive Performance Scale (CPS), a subscale from the RAI-MDS (version 2.1) [40,43]), use of physical restraints, psychotropic medication (classified according to the anatomical therapeutic chemical (ATC) classification system [44]), use of health care services (e.g. record of visits to the nursing home physician, psychologist and physiotherapist), comorbidity (classified according to classification of diseases in nursing home patients (CvZ-V) [45], compatible with the international classification of diseases, version 10 (ICD-10, [46]), dementia type and stage of dementia (Global Deterioration Scale (GDS)[29]). In addition, socio-demographic variables are assessed: gender, age, length of stay and living condition prior to admission.

#### Family caregivers

Primary outcome measures for family caregivers are: perceived burden, involvement with care and satisfaction with care provision. Perceived burden is measured with the 'Self-Perceived Pressure from Informal Care (SPPIC)' scale, a self-reported questionnaire consisting of 9 items [47,48]. Items are scored at a 5-point scale and form a one-dimensional Rasch scale, varying from less pressure to more pressure. Reliability (Rho = 0.79) and validity are found satisfactory for use in evaluation of intervention. Involvement with care is assessed by a self-report questionnaire, in which family caregivers report their frequency and length of visits, activities during a visit (based on the RAI-MDS subscale activities, version 2.1) and motivation for visiting. Satisfaction with care is assessed, using a self-reported questionnaire, which comprises 27 items, regarding care provided during the last 2–4 weeks. In addition, gender, age, relationship with the resident and sense of competence (Short Sense of Competence Questionnaire (SSCQ) [49]) are measured.

#### Nursing Staff

Job satisfaction and work motivation are the primary outcome measures for nursing staff. These are assessed using a self-reported questionnaire, consisting of 6 items [50]. Items are measured on a 5-point Likert scale, ranging from 1 'totally disagree' to 5 'totally agree'. Secondary outcome measures are: workplace social support (8-item scale from the Job Content Questionnaire [51,52]), job autonomy (Maastricht Autonomy Questionnaire [50]) and workload [50]. Finally, background variables age, gender, education level, contract working hours per week and employment years in institution type are recorded, as well as absentee rate.

#### Process evaluation

To investigate to what extent both types of dementia care settings are designed as they were intended, data is collected by researchers' observations and questionnaires at all three measurements. Observations regarding the selection criteria (e.g. joint household, staff tasks) are recorded in a logbook. The questionnaire comprises items relating to the organizational, social and physical environment of the unit and are measured at a 5-point Likert scale, ranging from 1 'not at all' to 5 'completely'. Item examples are: 'To what extent is nursing staff part of the household?' and 'To what extent resembles the design of the unit an archetype house?'.

To examine participants' satisfaction and experiences with small-scale living, self-report questionnaires (filled in by family caregivers and staff), are administered at the end of all measurements, i.e. T3. In addition, in-depth interviews are conducted with a selection of participants.

#### Procedure

Data from residents, family caregivers and nursing staff are collected at three moments a baseline measurement (T1) and 6 months (T2) and 12 months (T3) after baseline. Approximately 2 weeks prior to T1, a screening among residents is conducted to match residents at baseline (see Figure 1). The managing directors of the nursing homes and small-scale living facilities all provide consent to conduct the study. Written informed consent is obtained for all residents by their registered legal representative before participation. In addition, written informed consent is obtained for family caregivers and nursing staff in order to participate in the study.

#### Screening

The screening procedure to match residents at baseline, consists of 2 MDS subscales to assess cognition (CPS) and ADL-capacity (ADL-H) [40,41,43]. In addition, age, gender, length of stay and living condition prior to submission are measured. All residents in small-scale living facilities and psychogeriatric wards are assessed by the registered nurse (RN) of their unit. Cognition and ADL-scores are both dichotomized. Cut-off points are based on previous studies [38,53]. For cognition, the three lowest scores (i.e. 4, 5 and 6) are combined as a relatively low level of performance (category '-'); the remaining scores (i.e. 0 – 3) form a relatively high level of performance (category '+'). For ADL, the 4 lowest scores (i.e. 3 – 6) are considered as a relatively low level of functioning (category '-'). The other 3 scores (i.e. 0, 1 and 2) form a relatively high level of functioning (category '+'). Then, a cognition/ADL profile was constructed for each resident. Based on the profile of residents in small-scale living facilities, residents in psychogeriatric wards in traditional nursing homes with a relatively similar profile are recruited. This procedure is conducted to enhance comparability of groups at baseline with respect to cognition and ADL-capacity.

#### Data collection

The primary outcome measure for residents, quality of life (QUALIDEM), is assessed by 2 registered nurses (RNs) or certified nursing assistants (CNAs), as well as by residents' main family caregiver. Neuropsychiatric symptoms (NPI and CMAI), social engagement (ISE and RISE), ADL-capacity (ADL-H), cognition (CPS), use of physical restraints and use of services are assessed by RNs and CNAs. The nursing home physician or a psychologist administer the GDS, MMSE and CSDD. In addition, data regarding diagnosis and type of dementia, comorbidity and medication use are derived from medical records, as provided by the nursing home physician. Outcomes regarding family caregivers and nursing staff are based on self-report questionnaires.

### Sample size considerations

Sample size calculations are based on the primary outcome measure for residents, that is QoL, as measured by the QUALIDEM [26-28]. Using an effect size (δ) of 0.33, a significance level α of 0.05 (two sided) and a power of 90%, 84 residents are needed in each group. Based on previous research, the drop-out rate for residents in small-scale facilities appears to be lower than those in traditional nursing homes [17]. Taking these drop-out rates into account, we aim at including 120 residents in small-scale living facilities at baseline and 140 in traditional nursing homes to have a sufficient number of residents after 12 months (see also Figure 1).

### Statistical Analysis

Descriptive statistics are computed to describe background variables and characteristics of all participants, i.e. residents, family caregivers and staff. Baseline variables will be compared to investigate the comparability of residents at baseline. Multivariate regression analyses will be applied to estimate the differences in outcomes over time. Data will be analyzed according to the intention-to-treat principle, i.e. including all participants with valid data, regardless of whether they remained in the setting in which they were measured at baseline. In addition, on-treatment analyses will be performed, to investigate effects on participants who remained in the same care setting during all three measurements. In all analyses there will be correction for potential baseline differences. Drop-outs, relocations and losses to follow-up will be described. In addition, subgroup analyses will be performed to investigate participants' characteristics, who gain more benefits from small-scale living facilities than others. Data collected during the process evaluation will be mainly analyzed using descriptive techniques.

### Study Progress

Screening and inclusion of residents, family caregivers and professional caregivers started in April 2008 and will end in December 2008. Baseline measurements also started in April 2008. Follow-up measurements are planned for October 2008 – May 2009 and April – December 2009. In October 2008, baseline measurements have been performed for 106 residents living in small-scale living facilities and 93 residents living in psychogeriatric wards. In addition, 171 family caregivers are included (91 from small-scale living facilities and 80 from psychogeriatric wards) and 134 nursing staff members (71 in small-scale living and 63 from psychogeriatric wards). Dissemination of results is planned for 2010.

## Discussion

This paper presents the design of a longitudinal, quasi-experimental study to investigate the effects of small-scale living facilities for older people with dementia. Although some challenges concerning this design exist, it is currently the most feasible method to assess the effects of this relatively new dementia care setting.

Randomization in this study is difficult to realize due to ethical and practical drawbacks. Institutional care for people with dementia is seen in the Netherlands as a home for life. As a consequence, residents and their family members, together with clinicians, decide which accommodation suits their own wishes and beliefs best. This makes a random allocation of residents to a dementia care setting complicated, as seen in a study by Maas and Buckwalter (1990), reported in Saxton et al. (1998) [15,54]. Maas and Buckwalter tried to randomly assign residents to nursing home or special care unit, but family members had problems with accepting a random group allocation. In addition, it could take several years to acquire a moderate sample size of residents in small scale living facilities by using random assignment of residents. In the Netherlands, traditional nursing homes outnumber small scale living facilities and the latter seem to have a lower turnover rate [17], which makes random assignment difficult to realize.

To prevent selection bias, we have used a matching procedure in this study to enhance comparability of resident groups at baseline, with respect to cognition and ADL-capacity. We consider cognition and functional capacity as most important characteristics for matching, since these appear strongly related to dementia severity [55], especially discriminating between moderate and severe dementia [56]. A previous study has shown that residents living in small-scale living facilities had a higher cognitive and functional status at baseline, compared to those in regular psychogeriatric wards [17]. This emphasizes the need for creating comparable groups at baseline in order to study effects of the dementia care setting. Furthermore, the environment of both dementia care settings is well documented during the process evaluation, using registration, observation, questionnaires and in-depth interviews. As a result, differences and similarities between the two settings can be taken into account during the interpretation of results.

## Competing interests

The authors declare that they have no competing interests.

## Authors' contributions

All authors critically reviewed the manuscript, read and approved the final manuscript. HV, EvR, SMGZ, GIJMK and JPHH are involved in the study design. TA gave advices on the statistical analysis and sample size calculations.

## Pre-publication history

The pre-publication history for this paper can be accessed here:


